# Introducing melatonin to the horticultural industry: physiological roles, potential applications, and challenges

**DOI:** 10.1093/hr/uhac094

**Published:** 2022-04-22

**Authors:** Tengteng Gao, Xiaomin Liu, Kexin Tan, Danni Zhang, Bolin Zhu, Fengwang Ma, Chao Li

**Affiliations:** State Key Laboratory of Crop Stress Biology for Arid Areas/Shaanxi Key Laboratory of Apple, College of Horticulture, Northwest A&F University, Yangling 712100, Shaanxi, China; State Key Laboratory of Crop Stress Biology for Arid Areas/Shaanxi Key Laboratory of Apple, College of Horticulture, Northwest A&F University, Yangling 712100, Shaanxi, China; State Key Laboratory of Crop Stress Biology for Arid Areas/Shaanxi Key Laboratory of Apple, College of Horticulture, Northwest A&F University, Yangling 712100, Shaanxi, China; State Key Laboratory of Crop Stress Biology for Arid Areas/Shaanxi Key Laboratory of Apple, College of Horticulture, Northwest A&F University, Yangling 712100, Shaanxi, China; State Key Laboratory of Crop Stress Biology for Arid Areas/Shaanxi Key Laboratory of Apple, College of Horticulture, Northwest A&F University, Yangling 712100, Shaanxi, China; State Key Laboratory of Crop Stress Biology for Arid Areas/Shaanxi Key Laboratory of Apple, College of Horticulture, Northwest A&F University, Yangling 712100, Shaanxi, China; State Key Laboratory of Crop Stress Biology for Arid Areas/Shaanxi Key Laboratory of Apple, College of Horticulture, Northwest A&F University, Yangling 712100, Shaanxi, China

## Abstract

Melatonin (*N*-acetyl-5-methoxytryptamine) is an emerging biomolecule that influences horticultural crop growth, flowering, fruit ripening, postharvest preservation, and stress protection. It functions as a plant growth regulator, preservative and antimicrobial agent to promote seed germination, regulate root system architecture, influence flowering and pollen germination, promote fruit production, ensure postharvest preservation, and increase resistance to abiotic and biotic stresses. Here, we highlight the potential applications of melatonin in multiple aspects of horticulture, including molecular breeding, vegetative reproduction, production of virus-free plants, food safety, and horticultural crop processing. We also discuss its effects on parthenocarpy, autophagy, and arbuscular mycorrhizal symbiosis. Together, these many features contribute to the promise of melatonin for improving horticultural crop production and food safety. Effective translation of melatonin to the horticultural industry requires an understanding of the challenges associated with its uses, including the development of economically viable sources.

## Introduction

Horticultural crops include fruits, vegetables, flowers, medicinal and aromatic plants, and nuts and spices [1]. Horticultural crops are major sources of proteins, carbohydrates, vitamins, minerals, fiber, fats, micronutrients, and antioxidants in human nutrition. The world’s population is currently 7.7 billion and is projected to increase to 8.5 billion by 2030 and 9.7 billion by 2050 (United Nations, 2019). The shift towards population growth and healthier diets has led to an increased demand for horticultural crops. However, climate change and other abiotic and biotic stresses seriously affect horticultural production and will potentially increase in the future [2, 3]. The abuse of agrochemicals by growers in pursuit of high yields and profits negatively affects crop quality and food safety, and even threatens environmental and human health through the food chain [4]. The threats associated with chemical controls are increasing, and alternative eco-friendly protective agents must therefore be explored in order to achieve sustainable horticultural production.

Melatonin is a pleiotropic molecule with many diverse effects in living organisms. It has been one of the most intensively studied pleiotropic molecules in animals, showing many diverse effects on circadian rhythm, sleep, mood, retinal physiology, seasonal reproduction, and the immune system [5–9]. In plants, melatonin has been widely reported as a plant growth regulator (PGR) in seed germination, root proliferation, flowering, fruit set, and fruit ripening [10–13]. Furthermore, melatonin can extend the shelf life and maintain the quality of postharvest fruit [14]. Apart from affecting these normal physiological functions, melatonin is also known to function as an antioxidant, with important functions in scavenging both reactive nitrogen species and reactive oxygen species (ROS) [15–18]. Its ability to confer tolerance to various biotic and abiotic stresses, such as drought [19–21], high temperature [22], salinity [23, 24], cold [25], and microbial infections (fungi, bacteria, and viruses) [26–28] has been widely documented. In recent studies, melatonin has been used to reduce the accumulation of pesticide residues and heavy metals in foods [29, 30]. These features make melatonin an interesting candidate for improving horticultural crop production and ensuring food safety.

Here, we first review current applications of melatonin as an agrochemical (PGR, preservative, and antimicrobial agent) in horticultural crops. We then focus on its potential roles in the horticultural industry. Finally, challenges and future perspectives for the introduction of melatonin to the horticultural industry are proposed.

## Melatonin in plants: occurrence, biosynthesis, and perception

**Figure 1:**
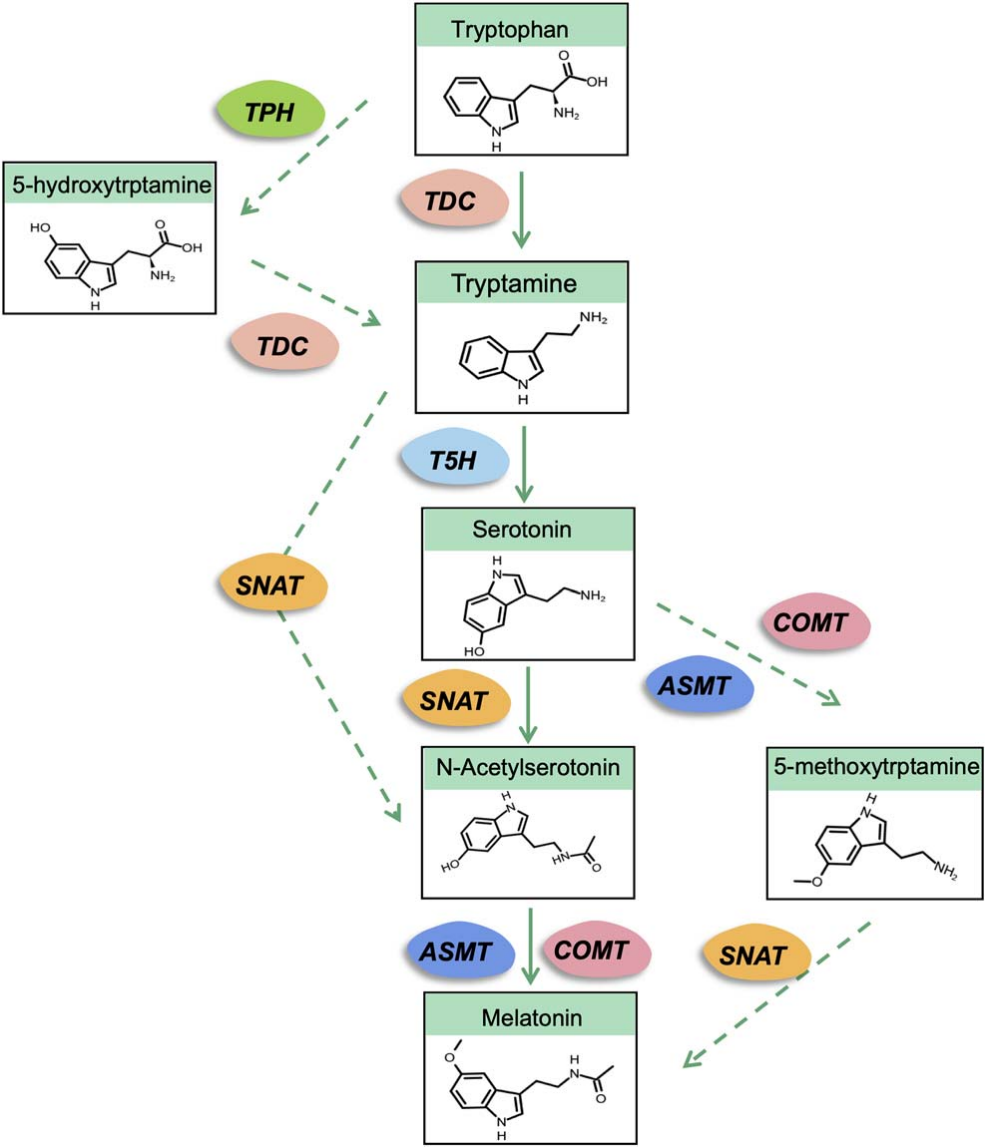
Melatonin biosynthesis pathways in plants. TDC, tryptophan decarboxylase; TPH, tryptophan hydroxylase; T5H, tryptamine 5-hydroxylase; SNAT, serotonin *N*-acetyltransferase; COMT, caffeic acid *O*-methyltransferase; ASMT, *N*-acetylserotonin methyltransferase.

Melatonin was first isolated from the pineal glands of bovine species in 1958 [31]. Thirty years after its discovery in mammals, the existence of melatonin in the unicellular dinoflagellate *Gonyaulax polyedra* completely challenged the concept that melatonin is an exclusive animal hormone [32]. It was not until 1995 that melatonin was detected simultaneously in vascular plants by two independent groups [33, 34]. Murch *et al*. [35] demonstrated that plants can synthesize melatonin though an isotope tracer study of *Hypericum perforatum* L. seedlings. Murch and Saxena [36] found that the biosynthetic pathway of melatonin in plants was analogous to that in animals, and both start from tryptophan. Based on published studies, the formation of melatonin starts from tryptophan and involves four successive enzymatic steps in plants [37] (Fig. 1). First, tryptophan is converted into tryptamine in a reaction catalyzed by tryptophan decarboxylase (TDC); the tryptamine is then changed into 5-hydroxytryptamine (serotonin) in the presence of tryptamine 5-hydroxylase (T5H) [38], and this is the major biosynthetic pathway of serotonin. In another biosynthesis pathway of serotonin, tryptophan is first converted to 5-hydroxytryptophan and then to serotonin by TDC [35]. *N*-Acetylserotonin is then generated from serotonin by the serotonin *N*-acetyltransferase (SNAT). Finally, melatonin is formed from *N*-acetylserotonin by the catalytic reaction of caffeic acid *O*-methyltransferase (COMT) or N-acetylserotonin methyltransferase (ASMT).

Melatonin plays a role in numerous plant growth and development processes, and is able to alleviate aspects of biotic and abiotic stress [10, 11, 22, 23, 26]. Because of its diverse biological functions, the term ‘phytomelatonin’ was proposed in 2004 [39]. More recently, Wei *et al*. [40] proposed that *Cand2* may act as a phytomelatonin receptor in *Arabidopsis thaliana*. *Cand2*/*PMTR1* was initially described as a plasma membrane protein with a receptor-like topology that interacts with the G-protein A subunit (GPA1); its expression in various organs and guard cells is tightly regulated by melatonin. Binding of melatonin to *Cand2* triggers the dissociation of Gα and Gγβ; this has been proposed to activate NADPH oxidase-dependent H_2_O_2_ production, promote K^+^ efflux and Ca^2+^ influx, and eventually lead to stomatal closure. Indeed, *Cand2* knockout mutants do not exhibit stomatal closure. In contrast to previous reports, Lee and Back [41] demonstrated that *Cand2* is not involved in melatonin-triggered defense gene induction, nor is *Cand2* located in the plasma membrane. Furthermore, the downstream Gα and Gβ subunits are not associated with the melatonin signaling pathway. In fact, many previous studies have indicated that melatonin application does not trigger the production of H_2_O_2_ in unstressed healthy plants [42–44], although a few studies show contradictory results [45, 46]. Thus, the application of melatonin in plants to produce H_2_O_2_ is still controversial and *Cand2* is considered to be a plant melatonin binding protein, not a melatonin receptor [47]. Further in-depth study of its signaling pathway and identification of its receptor is needed in order to fully realize the potential of melatonin. Although a melatonin receptor has not yet been definitely identified, many studies on its biological function support the use of melatonin in agriculture.

## Physiological roles of melatonin in horticultural crops: plant growth regulator, preservative, and antimicrobial agent

**Figure 2:**
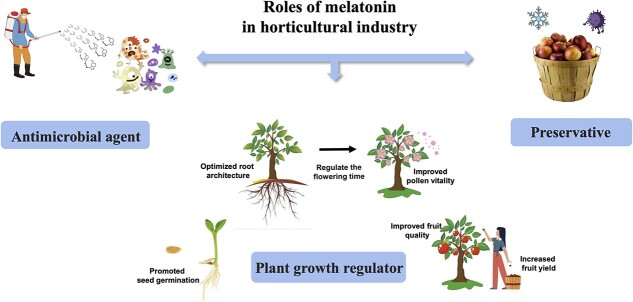
Physiological roles of melatonin in horticultural crops as a plant growth regulator, preservative, and antimicrobial agent.

Melatonin can be introduced to the horticultural industry by two different approaches: application as an agrochemical (Fig. 2) and the development of plant varieties that exhibit modified melatonin signaling or production. Field trials have validated the use of melatonin as an agrochemical on horticultural crops. Although some trials are still in the laboratory research stage, they have nonetheless demonstrated the potential of melatonin application for horticultural crops and have justified introducing melatonin to the horticultural industry.

### Plant growth regulator

#### Seed germination

Seed germination is a key process in the life cycle of plants and therefore has great significance for agricultural crop production [48]. Poor seed quality or unfavorable sowing conditions hinder seed germination and crop establishment and may eventually lead to yield loss [49]. Exogenous melatonin has been shown to affect seed germination under stress conditions. In field experiments, melatonin priming improved morphological traits, seed quality, and seed yield under water deficit in an arid cropping system [14]. During chilling stress, application of 25–100 μM melatonin to cucumber (*Cucumis sativus* L.) seeds also increased their germination rate and improved seedling growth and crop production [38]. Application of 20 μM melatonin improved seed germination under salt stress by promoting the accumulation of gibberellin (GA_3_) in cotton (*Gossypium hirsutum* L.) [50]. Under abscisic acid (ABA) stress, melatonin promoted the germination rate of melon (*Cucumis melo* L.) seeds by increasing the content of GA_3_ and reducing the content of ABA [51]. By contrast, Lv *et al*. [52] found that 10 or 100 μM melatonin did not affect seed germination, but 500 or 1000 μM melatonin significantly inhibited seed germination by increasing ABA and indole-3-acetic acid (IAA) levels under normal conditions. In this regard, it should be noted that melatonin has contrasting effects on seed germination under stress and normal conditions, perhaps because of cross-talk between melatonin and other hormones under different environmental conditions. Future studies should investigate the interactions between melatonin and plant hormones during seed germination after melatonin treatment.

#### Root system architecture

Adventitious rooting is a critical process of vegetative propagation for economically important horticultural crops. However, problems associated with rooting of cuttings cause frequent and significant economic losses [53]. Sarropoulou *et al*. [54] found that 1 μM melatonin enhanced the regeneration of adventitious root from shoot tip explants of cherry rootstock PHL-C (*Prunus avium* L. × *Prunus cerasus* L.). Melatonin treatment may increase rooting success rate and provide new possibilities for clonal propagation. Melatonin has been suggested to regulate root growth in a concentration-dependent manner, analogous to IAA [55, 56]. Pretreatment with low-concentration melatonin effectively promoted the formation and development of lateral roots, whereas high-concentration melatonin inhibited root growth [55, 56]. The application of 0.1 μM melatonin showed a stimulating effect on root growth in mustard (*Brassica juncea*) roots, whereas 100 μM had an inhibitory effect. Another study indicated endogenous levels of free IAA increased at low concentrations of melatonin, but there was no significant effect of treatment with high-concentration melatonin. The authors proposed that the stimulating effect of low-concentration melatonin on root growth was triggered by the biosynthesis of IAA stimulated by melatonin [56]. Pelagio-Flores *et al*. [57] used auxin-responsive marker constructs *BA3:uidA*, *DR5:uidA*, and *HS::AXR3NT-GUS* to investigate the effect of melatonin on auxin activity in *Arabidopsis* roots. In contrast to previous results, melatonin neither activated IAA to induce gene expression nor induced the degradation of *HS::AXR3NT-GUS*, indicating that melatonin may regulate root system architecture independently of IAA signaling in *Arabidopsis*. The above results show that the role of melatonin in plant growth and development is likely to be complex, and the IAA dependence or independence may depend on the process and plant under study.

#### Flowering and pollen germination

Flowering has a very important influence on harvest time and yield formation. The male fertility of crops can be inhibited under unfavorable conditions such as high temperature, resulting in pollen abortion. However, the inhibitory effect of high temperature on germination and viability of pollen was alleviated by applying 20 μM melatonin in tomatoes (*Solanum lycopersicum*) [22]. In this study, pollen abortion induced by high temperature was related to premature degeneration of tapetum cells and formation of defective pollen grains with degenerated nuclei. Pretreatment with melatonin increased the expression of autophagy-related genes (*ATG*s) and stimulated autophagosome formation. *ATG* mutants showed complete sporophytic male sterility due to pollen maturation defects and limited anther dehiscence in rice [58]. Melatonin degrades denatured proteins by enhancing the expression of *ATGs* and autophagosome formation, thereby restoring the stability of tapetum cells.

**Figure 3:**
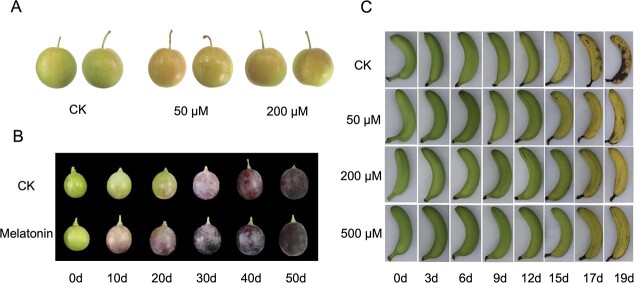
Exogenous melatonin promoted fruit coloring and delayed postharvest banana ripening. (A) Spraying 50 or 200 μM melatonin on red pear produced a markedly darker red color (Sun *et al*. [63]). (B) Grapes sprayed with 100 μM melatonin developed a darker color at maturity (Shen *et al*. [64]). (C) Melatonin treatment delayed postharvest banana ripening (Hu *et al*. [67]).

Fruit trees often encounter cold spells during flowering in the late spring. Delaying the flowering period of fruit trees can prevent freezing injury of flowers during such cold spells. Zhang *et al*. [59] monitored apple trees (*Malus domestica*) for two consecutive years and found that flowering was always related to the decline in melatonin level. Before the mixed buds germinated, ‘Fuji’ apple trees were sprayed with melatonin at different concentrations (0, 20, 200, and 1000 μM). The 20- and 200-μM melatonin treatments delayed apple bloom by 2 days relative to the control, and the 1000-μM melatonin treatment delayed bloom by 3 days [59]. In general, more flowers mean higher yields when water and fertilizer are sufficient [59]. Compared with the control (57.4% flowering rate), the 20- and 200-μM melatonin treatments improved the flowering rate to 63.8 and 72.7%, respectively. Thus, melatonin can be used to adjust the flowering period and extend the harvest period, suggesting new means of using melatonin to increase crop yields.

#### Fruit yield and quality

In previous studies, application of melatonin improved fruit yield [24, 25, 35, 36]. In field trials, treatment of pre-veraison grape berries (*Vitis vinifera* L.) with 100 mg l^−1^ (~431 μM) melatonin resulted in fruit that was ~6.6% heavier than control fruit [60]. Liu *et al*. [61] evaluated two application types of exogenous melatonin in tomato: root irrigation and seed-soaking. The yield of tomato plants irrigated with melatonin was increased obviously by 4%, and the fruit yield of seed-soaked plants was 13% higher. The authors therefore recommended soaking seeds with melatonin to improve fruit yields. Melatonin can significantly increase fruit yield even under biotic stress. Downy mildew caused by *Pseudoperonospora cubensis* is a significant threat to cucumber production. Pretreatment of cucumber seedlings with melatonin enhanced resistance to downy mildew and increased yields by 13.84–29.93% [62].

Exogenous melatonin can also increase fruit appearance and inner quality. In field trials, spraying 50 or 200 μM melatonin on red pear (*Pyrus ussuriensis*) during the pre-color-change period significantly increased anthocyanin concentration and produced a markedly darker red color [63] (Fig. 3A). Skin coloration is a major indicator of the commercial quality of red grapes. Field experiments revealed that the grape variety ‘Summer Black’ sprayed with 100 μM melatonin during veraison turned light red 10 days earlier, and the skin color of the berries at maturity was clearly darker than that of grapes without melatonin treatment [64] (Fig. 3B). The study also demonstrated that exogenous melatonin significantly improved the transcription level of anthocyanin biosynthesis genes and promoted anthocyanin accumulation in grape skin. Melatonin application also increased the content of soluble sugar and the concentrations of mineral nutrients in grape berries, such as N, K, Fe, Zn, and Cu [64]. Under long-term water deficit conditions, spraying 20 ppm (~86 μM) melatonin on tomato leaves significantly enhanced plant growth and improved fruit yield and quality, including the levels of ascorbic acid, total soluble solids, and lycopene [65].

However, the effects of exogenous melatonin treatment on fruit ripening are somewhat contradictory. In field trials, treatment with 10 μM melatonin promoted grape berry ripening by increasing ABA, H_2_O_2_, and ethylene production [66]. However, application of 10 μM melatonin delayed fruit ripening of sweet cherries (*Prunus avium* L.) by delaying anthocyanin accumulation and participating in cross-talk with cytokinins [12]. These results indicate that the same melatonin concentration may lead to different effects in different horticultural species.

### Preservative

Most postharvest fruits are extremely perishable, and cold storage is widely used to prevent the rotting of horticultural products and extend their shelf life [68, 69]. However, postharvest fruits are also susceptible to chilling damage when they are exposed to low temperatures and are vulnerable to diseases during storage. In recent years, melatonin has been assessed as a preservative to delay postharvest fruit senescence and quality deterioration while also enhancing chilling tolerance and disease resistance [70–72].

#### Ripening and senescence

Numerous studies have confirmed the effect of melatonin in the regulation of postharvest fruit ripening and senescence, especially for climacteric fruits. Postharvest treatment with melatonin reduced ethylene production in apple during postharvest storage [73]. Hu *et al*. [67] reported that melatonin treatment delayed postharvest ripening in banana (*Musa nana* Lour.), and this effect was concentration-dependent: 200 and 500 μM were more effective than 50 μM (Fig. 3C). Exogenous melatonin also reduced the production of ethylene by regulating the expression of *MaACO1* and *MaACS1* [73]. However, melatonin and ethylene do not necessarily act synergistically during the ripening process of postharvest fruits. For example, Sun *et al*. [74] found that 50 μM melatonin positively affected tomato fruit ethylene production, promoting its ripening. This result may be due to the complicated interaction between melatonin and ethylene or the temperature after harvest.

In ‘Kyoho’ grapes, the application of 200 μM melatonin reduced berry shedding and rot index by 37.50 and 58.37%, respectively [75]. Applying 100 μM melatonin delayed yellowing in broccoli (*Brassica oleracea* var. *italica*) by inhibiting the activities of chlorophyll catabolic enzymes and maintaining more intact chloroplasts during storage [69]. Melatonin also delayed softening of jujube fruit (*Ziziphus jujube* Mill.) by inhibiting the activities of cellulose, polygalacturonase, pectin methylesterase, and β-glucosidase [76]. Treatment of litchi (*Litchi chinensis* Sonn. cultivar ‘A4Wuhe’) with 400 μM melatonin alleviated pericarp browning by maintaining a higher fruit energy status, as indicated by increased ATP content and enhanced energy charge [77]. These results indicate that melatonin can delay postharvest fruit senescence by inhibiting energy metabolism and the activity of cell wall-degrading enzymes.

#### Chilling and disease resistance

Cold storage is often used to prevent rot and extend the shelf life of horticultural products. However, when fruit are exposed to low temperatures they are susceptible to chilling injury, which causes serious losses of quality and market value [78]. The green bell pepper, which is a subtropical fruit, is vulnerable to chilling injury under a cold environment. A previous study suggested that once the pepper fruit begins to show chilling injury, its physiological metabolism has become out of balance [79]. Kong *et al*. [71] found that treatment of bell pepper fruit (*Capsicum annuum*) with 100 μM melatonin reduced chilling injury by activating the antioxidant defense system and reducing cold-induced membrane lipid peroxidation during storage at 4°C. A previous study on ‘Baitangying’ litchi fruit treated with 400 μM melatonin (dipping for 20 minutes) showed that during shelf storage, by inhibiting the increase in malondialdehyde (MDA) content and the development of chilling injury, discoloration can also be inhibited, which may have been due to the enhancement of membrane integrity [80]. These results reveal that melatonin can inhibit the accumulation of ROS, preserving the structure and function of cells and tissues.

In recent years, the search for safe and effective alternative measures for disease control has attracted significant attention. Mandal *et al*. [72] applied melatonin to young cucumber fruits and tested the development of fruit rot after inoculation with *Phytophthora capsici*. They found that melatonin treatment significantly reduced both sporulation intensity and lesion diameter. Cherry tomatoes (*Lycopersicon esculentum* var. *cerasiforme*) were also soaked in 100 μM melatonin for 60 minutes, then stored at 22 ± 1°C; melatonin treatment significantly inhibited the development of gray mold induced by *Botrytis cinerea* through its effects on the phenylpropanoid pathway [81]. These results indicate that melatonin application has the potential to serve as an eco-friendly biocontrol approach for the prevention of postharvest disease.

### Antimicrobial agent

Infections by microbes (fungi, bacteria, and viruses), nematodes, and insects can cause serious losses in horticultural production. The most effective, rapid, and commonly used method for disease prevention is chemical control. However, the increasing demand for pesticide-free food crops and concerns about environmental sustainability have created a need for alternative, safer protective agents [82]. In this context, melatonin has shown promise as an antimicrobial agent against various diseases, with antifungal, antibacterial, and antiviral properties.

#### Antifungal agent

As an antifungal agent, melatonin has shown potential against potato late blight. *Phytophthora infestans* is the greatest threat to the production of potato (*Solanum tuberosum* L.) growth and has caused tremendous economic losses worldwide [83]. Exogenous melatonin may significantly inhibit the growth of mycelium, change the ultrastructure of cells, and induce the innate immunity of plants to pathogen infection. Fungicides and melatonin have shown synergistic antifungal effects: melatonin reduced the dosage levels and increased the efficacy of fungicide against *P. infestans* [26]. Application of 100 μM melatonin also enhanced the resistance of cucumber to downy mildew by improving its antioxidant ability and nitrogen metabolism capacity [57]. In contrast to these results, Lin *et al*. [84] reported that melatonin significantly decreased citrus fruit resistance to postharvest green mold. This result may be attributed to a decrease in H_2_O_2_ content and associated enzyme activity after melatonin treatment.

#### Antibacterial agent

Melatonin has shown effective antibacterial effects against plant bacterial pathogens. Melatonin treatment increased sugar and glycerol accumulation in response to infection by *Pseudomonas syringae* pv*. tomato* DC3000 (Pst DC3000). Raised sugars and glycerol then increased endogenous NO levels, thereby enhancing innate immunity against the pathogen through an NO- and salicylic acid-dependent pathway in *A. thaliana* [27]. Bacterial leaf streak of rice (*Oryza sativa* L.) caused by *Xanthomonas oryzae* pv. *oryzicola* also has a worldwide distribution. A previous study found that treatment with 200 mg l^−1^ (~861 μM) melatonin inhibited *X. oryzae* pv. *oryzicola* growth and reduced the bacterial population by 45% [85].

#### Antiviral agent

Compared with studies on bacterial and fungal disease, fewer studies have examined the effects of melatonin on plant viruses. However, many studies have suggested that melatonin can improve animal and human resistance to viral diseases, such as herpes simplex and Venezuelan equine encephalitis [86, 87]. Based on these studies, it has been hypothesized that melatonin application may also help to protect plants against viruses. Zhao *et al*. [28] applied exogenous melatonin (100 μM twice) to virus-infected tomato plants. The antiviral activity of these plants was enhanced, the virus titer and relative RNA levels of Tobacco mosaic virus (TMV) decreased, and the relative expression levels of *PR1* and *PR5* genes increased [28]. Melatonin-mediated plant resistance to viruses may represent a new approach for the control of viral diseases. Further studies are needed to investigate the effects of melatonin on plant–virus interactions.

## Potential applications of melatonin for the horticulture industry

**Figure 4:**
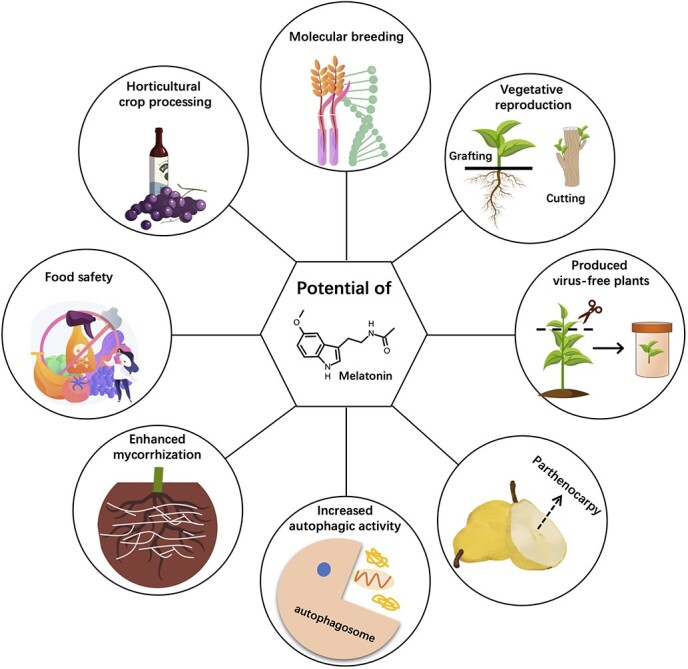
Potential applications of melatonin in the horticulture industry.

Recent studies have demonstrated numerous potential functions of melatonin in multiple aspects of horticulture, including molecular breeding [88–93], vegetative reproduction [94], virus-free plant production [95], food safety [30, 96–98], and horticultural crop processing [60, 99]. Melatonin can also influence key plant processes such as parthenocarpy [100], autophagy [42, 101, 102], and arbuscular mycorrhizal symbiosis [103, 104] (Fig. 4).

### Molecular breeding

Crop plants are often challenged by environmental stress, and genetic engineering and/or genetic transformation has opened up a new avenue for the genetic enhancement of complex abiotic and biotic stress tolerances. Using lines that overexpress melatonin biosynthetic enzymes to increase endogenous melatonin content can influence plant responses to various stresses by regulating the antioxidant system, promoting redox reactions, and inducing extensive transcriptional reprogramming. Transgenic *A. thaliana* plants that overexpressed apple *MzASMT1* exhibited significantly lower intrinsic ROS levels and greater drought tolerance than the wild type [88]. Under salt stress, transgenic *A. thaliana* overexpressing grape *VvSNAT1* showed enhanced growth and physiological performance, including reduced leaf wilting, longer root length, higher germination rate, and greater fresh weight [89]. The role of endogenous melatonin in biotic stress resistance has been investigated in previous studies. *A. thaliana* that overexpressed *ASMT* or *SNAT* showed upregulation of resistance gene expression (*PR1* and *PR5*), increased jasmonic acid (JA) content, smaller lesion size in leaves, and reduced plant disease symptoms [90].

In horticultural production, dwarfism plays a vital role in increasing planting density and improving yield per unit area. The use of dwarf rootstocks to permit intensive cultivation is the direction in which the modern fruit industry is developing. In previous studies, melatonin has shown the potential to control plant architecture and thus produce agricultural benefits. A semidwarf phenotype is commonly caused by deficiencies in melatonin synthesis and signal transduction; examples include the *COMT*, *TDC*, *SNAT2* RNAi, and *T5H* knockout rice lines and the *SNAT1* knockout and *Cand2* knockout *A. thaliana* mutants [91, 92]. In the orchard, seedlings with a better branching architecture are more easily formed into an ideal tree shape after planting, accelerating the time to productivity of a new orchard and promoting high yields. Wang *et al*. [93] found that transgenic ‘Micro-Tom’ tomatoes overexpressing the ovine (*Ovis aries*) melatonin synthesis genes *AANAT* (arylalkylamine *N*-acetyltransferase) and *HIOMT* (hydroxyindole-*O*-methyltransferase) had higher melatonin levels and lower IAA contents because melatonin and IAA share the same precursor, tryptophan. Therefore, both the *oAANAT* lines and the *oHIOMT* lines lost apical dominance and produced more branches.

A large number of transgenic plants with altered melatonin synthesis have been obtained, but there are few transgenic varieties of horticultural plants. Therefore, it will be important to design target products with high melatonin content by molecular breeding for melatonin biosynthesis genes in different horticultural species, including trees, vegetables, and flowers.

### Vegetative reproduction

Vegetative reproduction is an asexual method of plant propagation in which new plants are produced from vegetative parts such as leaves, stems, and roots [105]. However, plants are often subjected to many environmental stresses during the process of vegetative reproduction, resulting in economic losses.

Chinese hickory (*Carya cathayensis*), which is popular for its nuts, is an economically important tree species [106]. However, it requires a long period to reach the nut-producing stage, and grafting is therefore widely used to accelerate its production [107]. Water conductance plays a crucial role during grafting, and water scarcity would threaten graft survival during the process of graft union. Sharma *et al*. [94] showed that the application of melatonin successfully promoted the growth of grafted plants by boosting the antioxidant defense system, improving photosynthetic efficiency, and triggering the accumulation of compatible solutes under drought stress. Watermelon (*Citrullus lanatus*) is vulnerable to cold stress, and grafting it onto pumpkin can enhance its cold tolerance [108]. In recent studies, melatonin application to pumpkin or fig leaf gourd rootstock conferred cold tolerance to the watermelon shoots. The application of melatonin to the roots promoted melatonin accumulation in leaves and induced the accumulation of methyl jasmonate. Methyl jasmonate then triggered H_2_O_2_ production and enhanced watermelon shoot cold tolerance [25].

Cutting propagation is one of the most common methods for vegetative reproduction of a large number of horticultural plants, including *Vitis*. Cultivated primarily in arid or semi-arid regions, grape is often vulnerable to drought stress, and the development of effective drought-resistant cultivation strategies is therefore critical for viticulture. A previous study found that application of melatonin to the roots of *V. vinifera* cuttings improved their resistance to drought stress simulated by polyethylene glycol [60]. After 12 days, most seedling leaves turned yellow, became blighted, or dropped off in the absence of melatonin application. By contrast, application of 0.05, 0.1, or 0.2 μM melatonin successfully alleviated the stress damage to some extent. Application of 0.1 μM melatonin reduced the drought index from 65.4% in the control to 37.1% in the treated plants. These results indicate that application of melatonin can effectively alleviate abiotic stress and promote the growth of crops produced from cuttings and grafted plants.

### Production of virus-free plants

Virus infection can cause graft incompatibility, reduce the rooting of cuttings, decrease photosynthesis and vegetative growth, and result in yield losses [109]. Some horticultural plants, such as apples, are always propagated vegetatively by grafting and are thus vulnerable to virus transmission. Cultivation of virus-free plants is a common strategy for efficient and economical control of apple viral diseases [110].

Apple stem grooving virus (ASGV) is a serious virus that attacks apple and has occurred in many areas around the world [111]. In previous studies, Chen *et al*. [95] examined the ability of exogenous melatonin application to eradicate ASGV from infected *in vitro* shoots. Application of 15 μM melatonin increased the number of shoots and the shoot length and promoted shoot regrowth levels in the shoot tips of infected cultured shoots. After 4 weeks of proliferation, the shoot tips of infected *in vitro* shoots cultured on medium with melatonin were 95% virus-free, whereas no virus-free shoots were produced when infected shoots were cultured without melatonin. Therefore, melatonin treatment provides a novel way to eradicate plant viruses and shows great potential in producing virus-free plants.

### Parthenocarpy

Parthenocarpy, which means the production of seedless fruit without fertilization, could be genetic (occurring autonomously) or artificial (stimulated) [112]. The main advantage of parthenocarpy is that fruit set and yield are not suppressed by environmental conditions that are unfavorable to pollination and fertilization [113]. Moreover, the absence of seeds increases the fruit quality of many horticultural plants since seeds usually have an unpleasant taste and are difficult to digest. Parthenocarpy can be induced by applying plant hormones, such as GAs [114]. In ‘Cuiguan’ pear (*Pyrus pyrifolia* Nakai), GAs induced the development of high-quality parthenocarpic fruits with a high edible rate and small cores [115]. In previous studies, melatonin has been shown to regulate GA synthesis and stabilize the GA downstream inhibitor DELLA proteins [116]. Thus, melatonin may cause parthenocarpy by regulating GA pathways. Liu *et al*. [100] found that exogenous melatonin induced parthenocarpy in ‘Starkrimson’ pear and significantly increased GA biosynthesis by regulating the expression of *GA20ox* and *GA2ox*. Therefore, melatonin induces parthenocarpy in pear by promoting GA biosynthesis.

### Autophagy

Autophagy, an intracellular degradation system that delivers proteins and organelles to lysosomes, is involved in numerous biological processes of plants, such as seedling establishment, root meristem maintenance, male fertility, pollen germination, senescence, and responses to biotic and abiotic stress [117–119]. Previous studies have found that melatonin could improve resistance to abiotic stress by regulating autophagic activity [42, 101, 102].

Tomato plants pretreated with 10 μM melatonin and *ASMT*-overexpressing tomato plants showed higher expression of six autophagy-related genes (*ATG5*, *ATG6*, *ATG8a*, *ATG8f*, *ATG12*, and *ATG18c*) and higher numbers of autophagic signals compared with wild-type plants under heat stress [120]. *A. thaliana* pretreated with 5 or 10 μM melatonin exhibited higher levels of autophagy and increased resistance to methyl viologen (MV)-induced oxidative stress [42]. Furthermore, transgenic *A. thaliana* overexpressing the alfalfa *MsSNAT* gene showed greater salt tolerance through an increase in autophagy, manifested in increased autophagosome numbers and upregulation of some autophagy-related genes [101].

In recent studies, *MeASMT2*, *MeASMT3*, and *MeTDC2* have been shown to actively regulate autophagic activity in cassava. Overexpressed *MeATG8b*, *8c*, and *8e* also promoted the expression levels of *MeASMT2*, *MeASMT3*, and *MeTDC2 in vivo*. Further investigation showed that *MeATG8b*/*8c*/*8e* interacted with *MeASMT2*, *MeASMT3*, and *MeTDC2* [121]. To date, the direct connection between melatonin and autophagy and its underlying mechanism remain elusive. However, these findings expand our understanding of the coordination between autophagy signaling and melatonin synthesis in plants, and they will help us to improve the resistance of plants by exploiting the synergistic effects of melatonin and autophagy in the future.

### Arbuscular mycorrhizal symbiosis

Arbuscular mycorrhizal fungi (AMF), a type of beneficial soil microorganism, form symbioses with almost 80% of land plants, including most agricultural crop species [122]. AMF obtain photosynthetic products from the host plant and in turn provide water and mineral nutrients to the host [123, 124]. In recent studies, 100 μM melatonin treatment increased the AMF colonization rate in cucumber plants. Importantly, synergism between AMF and melatonin enhanced resistance to fusarium wilt compared with either treatment alone [103]. Liu *et al*. [104] also reported that treatment with exogenous melatonin significantly promoted arbuscular mycorrhizal symbiosis in tobacco seedlings under drought conditions. In addition, the combined application of melatonin and AMF had an additive effect, making plants more tolerant to drought stress and more productive. The role of melatonin signaling in arbuscular mycorrhizal symbiosis is emerging as an interesting area of research and has implications for plant growth, water and nutrient absorption, and stress resistance.

### Food safety (heavy metals and pesticide residues)

Heavy metals in soils are derived from the weathering of soil minerals, the application of sewage sludge, the use of fertilizers with high heavy metal contents, and industrial activities [125, 126]. These metals enter food chains through plant uptake and cause serious harm to humans and animals [127]. Melatonin treatment reduced oxidative stress and limited the translocation of cadmium to shoots of mallow (*Malva parviflora*) exposed to cadmium [96]. Melatonin also lowered vanadium concentrations in leaves and stems by reducing vanadium transport from roots to shoots in watermelon [29]. Foliar application of 100 μM melatonin dramatically reduced leaf arsenic content in the ‘Longjing 43’ tea cultivar [97]. These results illustrate that melatonin can be applied to reduce the availability of heavy metals to plants, thereby decreasing their accumulation in edible parts.

Overuse of pesticides by growers in pursuit of yields and profits also has a negative impact on crop quality and food safety, even threatening environmental and human health by introducing pesticides to the food chain [4, 128]. Treatment with 100 μM melatonin reduced the pesticide content of jujube fruit by ~85, 60, and 44% when applied with chlorothalonil, malathion, and glyphosate, respectively, after 5 days of storage [98]. Application of 500 μM melatonin also decreased residues of the fungicide carbendazim in the leaves of spinach (*Spinacia oleracea* L.), lettuce (*Lactuca sativa* L.), Chinese cabbage (*Brassica campestris* L.), celery (*Apium graveolens* L.), melon, and cucumber by an average of 49–54% [30]. Furthermore, overexpression of the melatonin biosynthesis gene *COMT1* significantly increased melatonin biosynthesis, thereby reducing fungicide phytotoxicity and residues in tomato plants [30]. More importantly, a grafting experiment showed that the use of *COMT1* transgenic lines as rootstocks not only produced melatonin-enriched fruit but also alleviated pesticide phytotoxicity and residues through root-sourced melatonin signaling. Therefore, more attention should be focused on plant pesticide metabolism, using both exogenous chemical approaches and transgenic methods in order to solve food safety problems.

### Horticultural crop processing

The melatonin content in horticultural products is also affected by processing techniques. Kirakosyan *et al*. [129] compared the content of melatonin in frozen cherries, dried cherries, cherry juice, and powders made from individually quick-frozen cherries. They did not detect melatonin in dried cherries or cherry juice, but it was detected in frozen cherries and cherry powders, and the melatonin content of frozen cherries was significantly higher than that of powders. These results may reflect the fact that melatonin is unstable and easily degraded during processes that destroy cell structure. During tea processing, heat treatment reduced the melatonin content of mulberry leaves (cultivar ‘Buriram 60’) by ~87% compared with fresh leaves. On the contrary, no significant difference was detected between melatonin contents of mulberry leaf tea produced with and without blanching (i.e. green tea and black tea). Melatonin content of mulberry was highest in the leaf tips, followed by young leaves, and lowest in old leaves [130]. Therefore, cultivar selection and leaf age are two additional factors to consider when using mulberry leaves. The optimal conditions should be studied for processing mulberry leaf tea in order to maximize the yield of melatonin in final products.

Melatonin can be synthesized during the winemaking process, especially after alcoholic fermentation [131]. The composition of volatile compounds in wine produced from berries treated with melatonin was significantly different from that in wine produced from untreated berries. Exogenous melatonin application increased spicy, sweet sensory, and fruity properties in wines. These effects could be enhanced by prolonging the treatment through repeated treatment, and more pronounced effects on wine aroma characteristics were observed [60]. In addition, Xu *et al*. [99] found that treatment of veraison grape berries with melatonin promoted the antioxidant capacity of wine and increased the contents of flavonoids, anthocyanins, and total phenols. These results highlight the potential for melatonin application to improve red wine quality.

## Challenges

For effective translation of melatonin to the horticultural industry, it will be critical to gain a better understanding of several challenges associated with its use, including the identification of effective melatonin concentrations and the development of economically viable melatonin sources.

### Effective concentration, application method, and processing time

The optimal melatonin dosage, application method, and duration appear to differ among different species and plant parts. Three application parameters should be considered in order to apply melatonin effectively: concentration, method, and processing time.

The biphasic dose–response effect of melatonin on plants is very important for its horticultural applications; maximum enhancement of plant response occurs within a specific dose range, beyond which melatonin may have reduced or even negative effects. Many studies have found that melatonin has a hormetic dose–response relationship (i.e. low-dose stimulation and high-dose inhibition). Hernández-Ruiz *et al*. [55] found that melatonin, like IAA, promotes vegetative growth in etiolated *Lupinus albus* L. hypocotyls. It promotes growth in the micromolar concentration range with an optimal concentration of 10 μM, and it inhibits growth at higher concentrations. Similarly, previous studies have demonstrated that melatonin promotes rooting at a low concentration but inhibits it at high concentrations [132]. These results indicated that melatonin may exhibit a hormetic–biphasic effect characteristic of hormones. As shown in Table 1, the effective concentration range of 1–1000 μM has been used in many studies.

**Table 1:** Summary of recent reports on melatonin dosage, application method, and duration for horticultural crops

**Plant species**	**Treated plant tissues**	**Plant responses**	**Application method**	**Effective concentration (**μM**)**	**Processing time**	**References**
*Actinidia chinensis* Planch	Root	Improved drought resistance	Irrigated	100	8 days (at 2-day intervals)	Liang *et al*. [133]
	Root	Improved heat resistance	Irrigated	200	10 days (at 2-day intervals)	Xia *et al*. [134]
*Brassica oleracea var. italica*	Florets	Increased glucosinolate contents	Immersed	1	5 minutes	Miao *et al*. [135]
	Florets	Positively affected the glucoraphanin-sulforaphane system	Immersed	100	10 minutes	Wei *et al*. [136]
*Brassica rapa* ssp. *parachinensis*	Cabbages	Maintained postharvest quality	Immersed	100	3 minutes	Tan *et al*. [137]
*Camellia sinensis* L.	Leaves	Improved arsenic tolerance	Sprayed	100	2 times (once every 3 days)	Li *et al*. [97]
	Shoot	Alleviated cold stress	Sprayed	100	3 times (once every 5 days)	Li *et al*. [45]
*Carya cathayensis* Sarg.	Shoot	Improved drought resistance	Sprayed	100	5 days (once per day)	Sharma *et al*. [94]
*Capsicum annuum*	Seeds	Alleviated herbicide and drought stresses	Immersed	50	6 hours	Kaya *et al*. [138]
	Fruits	Improved cold resistance	Immersed	100	30 minutes	Kong *et al*. [71]
*Cucumis sativus*	Root	Resisted downy mildew	Irrigated	100	2 times (once every 3 days)	Sun *et al*. [62]
	Leaves	Increased AMF-Induced resistance to *Fusarium* wilt	Sprayed	100	At 5-day intervals	Ahammed *et al*. [103]
*Cucumis melo* L.	Seeds	Enhanced salt tolerance	Immersed	50	6 hours	Xiong *et al*. [139]
	Seeds	Enhanced copper tolerance	Immersed	100	12 hours	Hu *et al*. [140]
	Seeds	Promoted root development under copper stress	Immersed	100	12 hours	Hu *et al*. [140]
*Eriobotrya japonica* Lindl.	Root	Improved drought resistance	Irrigated	150	15 days (once every 3 days)	Wang *et al*. [141]
*Fragaria ananassa* Duch.	Leaves	Alleviated cadmium stress	Sprayed	100	3 times (once every 2 days)	Wu *et al*. [142]
.	Fruits	Improved cold resistance	Immersed	100	5 minutes	Aghdam and Fard [143]
	Fruits	Delayed rotting and improved quality	Immersed	50 or 100	30 minutes	Pang *et al*. [144]
*Gossypium hirsutum* L.	Leaves	Improved male fertility	Sprayed	100 or 200	/	Hu *et al*. [145]
	Seeds	Promoted seed germination	Immersed	20	24 hours	Chen *et al*. [50]
*Litchi chinensis* Sonn.	Fruits	Alleviated pericarp browning	Immersed	400	5 minutes	Wang *et al*. [77]
	Fruits	Increased resistance to downy blight	Immersed	250	15 minutes	Zhang *et al*. [146]
	Fruits	Delayed senescence and pericarp browning	Immersed	400	5 minutes	Zhang *et al*. [147]
*Malus pumila* Mill.	Leaves	Delayed flowering and resulted in more flowering	Sprayed	20, 200, or 1000	From 25 March to 12 April (once every 2 days)	Zhang *et al*. [59]
	Fruits	Delayed postharvest ripening and improved appearance quality	Sprayed	100	Once (postharvest)	Onik *et al*. [73]
	Root	Improved drought resistance	Irrigated	100	60 days (once every 10 days)	Liang *et al*. [148]
*Mangifera indica* L.	Fruits	Delayed ripening and softening	Immersed	500	1 hour	Liu *et al*. [149]
*Musa acuminata* L.	Fruits	Delayed postharvest ripening	Immersed	200 or 500	2 hours	Hu *et al*. [67]
	Fruits	Enhanced chilling tolerance and alleviated peel browning	Immersed	200	2 minutes	Wang *et al*. [150]
*Nicotiana tabacum* L.	Leaves	Enhanced the AM colonization rate	Sprayed	200	30 days (once every 4 days)	Liu *et al*. [104]
*Prunus salicina* L.	Fruits	Improved cold resistance	Immersed	100	100 minutes	Du *et al*. [151]
*Pyrus communis* L.	Fruits	Limited softening and reduced physiological disorder	Immersed	100	12 hours	Zhai *et al*. [152]
	Flowers	Induced parthenocarpy	Sprayed	100	At anthesis	Liu *et al*. [100]
*Prunus avium* L.	Fruits	Delayed fruit ripening	Sprayed	10	1 time	Tijero *et al*. [12]
*Solanum lycopersicum*	Root	Improved fruit quality	Irrigated	100	From fruit set to maturity (once a week)	Liu *et al*. [61]
	Fruits	Promoted ripening and improved quality	Immersed	50	2 hours	Sun *et al*. [74]
	Root	Alleviated high temperature-induced pollen abortion	Irrigated	20	7 days	Qi *et al*. [22]
	Fruits	Improved cold resistance	Immersed	100	5 minutes	Sharafi *et al*. [153]
	Seeds	Increased fruit yield	Immersed	100	5 hours	Liu *et al*. [61]
	Fruits	Enhanced disease resistance	Immersed	100	1 hour	Li *et al*. [81]
	Fruits	Promoted carotenoid biosynthesis	Immersed	50	2 hours	Sun *et al*. [74]
*Vicia faba*	Root	Enhanced arsenic resistance	Irrigated	50	34 days (once every 3 days)	Siddiqui *et al*. [154]
*Vitis vinifera* L.	Fruits	Improved cold resistance	Immersed	200	25 minutes	Wang *et al*. [75]
	Grape cluster	Promoted ripening	Immersed for 5 s	100	2 times (at 46 and 53 days after bloom)	Xu *et al*. [66]
	Grape cluster	Alters secondary metabolite accumulation	Immersed for 5 s	50	At 70 days after full bloom	Ma *et al*. [155]
*Ziziphus jujuba* Mill.	Fruits	Improved storage quality	Immersed	25	2 minutes	Tang *et al*. [76]

Previous studies have used three main application methods: spraying, irrigation, and soaking. Spraying is mainly aimed at leaves, irrigation at roots, and soaking at seeds and fruits. Different organs and tissues have been found to respond differently to melatonin. Therefore, the application method should be tailored to the plant species, rate of uptake, transport to target tissue, and intended purpose. In the spraying method, melatonin is sprayed uniformly onto the plant tissues and is then absorbed by the plant. Uniform application of the material to the tissue is essential, as missing some portions of the plant may affect its morphological characteristics. No matter which application method is used, melatonin solutions should be applied toward evening to avoid any light degradation of melatonin and achieve the best results.

Treatment time is also an important melatonin application parameter. In previous studies, the duration of melatonin treatment has differed among plant species, tissues, and purposes (Table 1). Stress tolerance can be maximized by studying dose–time response relationships in order to determine the minimum dose and duration required to induce the maximum biological response. In the future, the concentrations and durations of melatonin treatment will require further refinement to shorten the treatment time.

The positive effects of melatonin as a PGR are significantly affected by its concentration, and concentration must be adjusted based on plant type, application period, location of application, and application method. The best melatonin concentration for a specific type of application and species must be determined if melatonin is to become a widely used agricultural product.

### Production of melatonin

Although melatonin has been explored as an agricultural chemical in field trials, it is rarely used in agricultural production because of its high cost. Melatonin was first extracted from the pineal glands of animals [31]. However, this extraction process poses many challenges, such as limited sources, high cost, low output, and the risk of viral infection.

**Figure 5:**
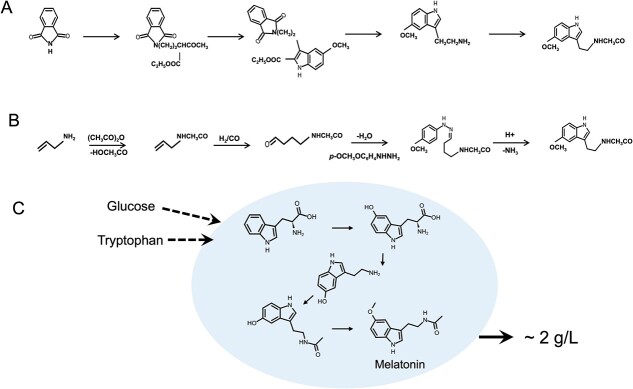
Chemical synthesis and microbial synthesis of melatonin. The biosynthesis pathway of melatonin as reported by He *et al*. [156] (A) and Verspui *et al*. [157] (B). (C) The melatonin biosynthesis pathway constructed in *Escherichia coli*.

Currently, industrial production of melatonin is performed mainly by chemical synthesis. Figure 5 shows one of the commonly used chemical synthesis strategies; it requires toxic substrates or catalysts, including *p*-anisidine and 1,3-dibromopropane [156] (Fig. 5A). Another commercial melatonin synthesis method makes use of the Fischer indole reaction starting from allylamine [157] (Fig. 5B). By successive acetylation of allylamine and selective hydroformylation, 4-acetamidobutanal is produced and reacted with 4-methoxyphenylhydrazine. This synthesis is performed in one vessel, and 44% melatonin yield can be achieved. However, the starting material, allylamine, has few sources; it is expensive, extremely flammable, highly toxic, and a strong irritant. Therefore, there is a great need for safer and more cost-effective strategies for industrial-scale melatonin production.

Recent studies suggest that microbial production is a possible method for more effective, safer synthesis of higher melatonin quantities. Germann *et al*. [158] constructed a recombinant melatonin pathway in the yeast *Saccharomyces cerevisiae* and achieved *de novo* biological production of melatonin directly from glucose. In their study, the cells produced 0.3–0.8 mg l^−1^ of melatonin from glucose after the introduction of eight melatonin biosynthesis genes and cofactor supporting pathways. In a 76-hour fermentation using glucose as the sole carbon source, the supply of acetyl-CoA was enhanced by overexpressing acetaldehyde dehydrogenase and boosting the supply of this cofactor, eventually increasing the production of melatonin to 14.50 ± 0.57 mg l^−1^. Similarly, Hao *et al*. [159] reported the biosynthesis of melatonin at a high titer using *Escherichia coli* recombinant cells (Fig. 5C). They initially produced 0.13 g l^−1^ of melatonin from tryptophan under batch-fed fermentation conditions. After fermentation optimization and engineering modifications, the maximum melatonin titer reached 1 g l^−1^ with glucose as the sole carbon source, and 2 g l^−1^ with external supplementation of tryptophan. These studies make a compelling case for microbial synthesis, and this approach could potentially be the foundation for commercial melatonin production using microbial cell factories in the near future.

## Future perspectives and conclusions

The use of melatonin as a novel eco-friendly agrochemical and the development of new crop varieties have great potential to meet agricultural challenges by regulating plant growth, extending the preservation of postharvest fruits, and increasing resistance to abiotic and biotic stresses.

PGRs are widely used in horticultural crops; they can stimulate flowering, promote fruit ripening, and improve fruit quality. Global Market Insights reported that the global PGR market exceeded US$5 billion in 2017 and is expected to reach ~US$10 billion by 2025. Natural and synthetic auxins are used to stimulate rapid and prolific rooting and thin flowers or fruits, and improve fruit setting or preharvest fruit retention. Some climacteric fruit, such as avocados (*Persea americana* Mill.), bananas, kiwifruits (*Actinidia deliciosa* cultivar ‘Bruno’) and mango (*Mangifera indica* L.), must be harvested before the climacteric phase and artificially ripened with ethephon to extend their shelf life [160]. Like other PGRs, melatonin is expected to be used to improve the agronomic properties of horticultural plants.

Semidwarfing varieties of wheat and rice are key promoters of the green revolution, which has triggered extraordinary increases in crop productivity [161]. In breeding programs, transgenic crop varieties have shown good prospects for the control of plant architecture. The relationship between semidwarfing and phytohormones has been studied extensively in various crops. For example, mutations associated with brassinosteroids can cause semidwarfism, and the barley (*Hordeum vulgare* L.) *uzu* mutation has been introduced into commercial varieties to produce lodging-resistant, high-yielding varieties [162]. Strigolactones (SLs) are a class of phytohormones that inhibit shoot branching in plants. Loss of function of the SL biosynthesis gene *CCD7* (carotenoid cleavage dioxygenase) contributed to increased tiller number and improved grain yields in rice. Some melatonin-related research has shown that semidwarf seedling is a common phenotype of melatonin-deficient plants. *TDC* RNAi rice exhibited a semidwarf phenotype compared with the wild type. *T5H* knockout rice and *COMT* RNAi rice had lower *DWARF4* gene expression and lower brassinosteroid levels, which produced a semidwarf, erect-leaf phenotype [91]. In addition, *oAANAT* and *oHIOMT* overexpression lines lost apical dominance and exhibited more branches in tomato [93]. In future plant breeding, genetic approaches that regulate melatonin biosynthesis may also be used to increase yield. However, it is important that these methods promote improved crop architecture while minimizing any negative traits associated with the relevant signaling pathways.

In human health, melatonin has shown beneficial effects on sleep disturbances [163], exhibits antioxidant capacity [164] and antidiabetic properties [165], and helps with the treatment of autism spectrum disorder [166]. Melatonin ingested in food can enter the blood and bind to melatonin binding sites in the mammalian brain [34]. Dubbels *et al*. [33] reported that the consumption of plant products with a high melatonin content can change the melatonin level in blood and protect macromolecules from oxidative damage. Melatonin not only has health functions itself but can also improve the accumulation of other beneficial substances, such as anthocyanins and phenols [75, 167–172]. Anthocyanins are bioactive compounds of considerable interest due to their antioxidant and anti-inflammatory properties [168, 169]. Phenolic compounds also have important antioxidant properties and are thought to exert protective effects against cardiovascular and neurodegenerative diseases [170–172]. In addition, 1 μM melatonin promoted a high content of glucosinolates and resulted in an increase in the percentage of glucoraphanin, an effective anti-cancer component [173]. Exogenous melatonin treatment and genetically modified plants could increase the endogenous melatonin in horticultural products. Fruit intake may affect the level of endogenous melatonin in the human body. However, the optimal dosage and timing of melatonin administration are unclear. There is also potential public resistance to the fact that the melatonin concentration applied in most studies exceeds the physiological levels present in plants. Although short-term human exposure appears to be relatively safe, the effects of long-term exposure are unknown. Therefore, the effects of melatonin on human health require further study.

In conclusion, numerous examples in horticultural crops have demonstrated that the development of new crop varieties and the use of melatonin as a PGR, preservative, and antimicrobial agent have great potential to address grand challenges in the horticultural industry. Field trials have shown the promise of melatonin for regulating fruit set and fruit ripening, promoting fruit postharvest preservation, and improving abiotic/biotic stress resistance. However, melatonin has significant untapped potential, and many of its possible benefits to the horticultural industry remain unclear. Therefore, potential applications of melatonin should be tested through field trials. Less expensive sources of melatonin are also required. In the future, the development of crop varieties with increased melatonin production or modified melatonin signaling will undoubtedly give rise to new paradigms for the application of melatonin in horticultural crops in a changing environment.

## Acknowledgements

This work was supported by the National Natural Science Foundation of China (31972389), the earmarked fund for the China Agricultural Research System (CARS-27), and the National Key Research and Development Program of China (2018YFD1000303).

## Author contributions

F.M. conceived the study; T.G. and X.L. wrote the manuscript; K.T., D.Z., and B.Z. contributed to collection and sorting of the references. C.L. revised the manuscript. All authors read and approved the final manuscript.

## Conflict of interest

The authors declare no conflicts of interest.
